# A Review of Friction in Low-Stress Mechanics of Fibrous Flexible Materials

**DOI:** 10.3390/ma17153828

**Published:** 2024-08-02

**Authors:** Liang Luo, George Stylios

**Affiliations:** Research Institute for Flexible Materials, Heriot Watt University, Edinburgh EH14 4AS, UK

**Keywords:** fabric friction, low-stress mechanics, fabric hysteresis, elastic and frictional fabric behaviour, friction models

## Abstract

The structure of a fabric is a highly complex assembly of fibres, which have order and regularity as well as disorder and randomness. The complexity of the structure poses challenges in defining its mechanical behaviour, particularly at low stress, which is typical to end uses. The coexistence of multiple deformations and the high degree of nonlinearity of the fabric due to fibre friction make its stress–strain relationship complicated. This article reviews the literature on friction related to the low-stress mechanics of fabrics, and it establishes its range and regularity to help with finding a unified reference model, in which although the physical meanings of fabric tensile, shear, and bending vary, they follow consistent mathematical regularities. So, invariably, their disorder and randomness needed in defining them can be obtained from fabric measurement data. It defines the scope and patterns of friction to facilitate the development of a unified reference model. It argues that although the physical interpretations of fabric tensile, shear, and bending characteristics may differ, they adhere to consistent mathematical regularities within this model, and hence extracting disorder and randomness from fabric measurement data may be achievable. This paper concludes with a number of recommendations, postulating that hysteresis caused by friction between fibres in a fabric is an important component of mechanical information, and it coexists with its purely elastic component, but it cannot be obtained directly by measurement. Seeking means to effectively decompose the friction hysteresis and pure elastic components contained in fabric mechanics measurement data will provide an accurate characterization of fabric mechanical properties and hence an accurate modelling and simulation of its behaviour, and will impact many traditional and industrial textile end uses.

## 1. Introduction

**Figure 1:**
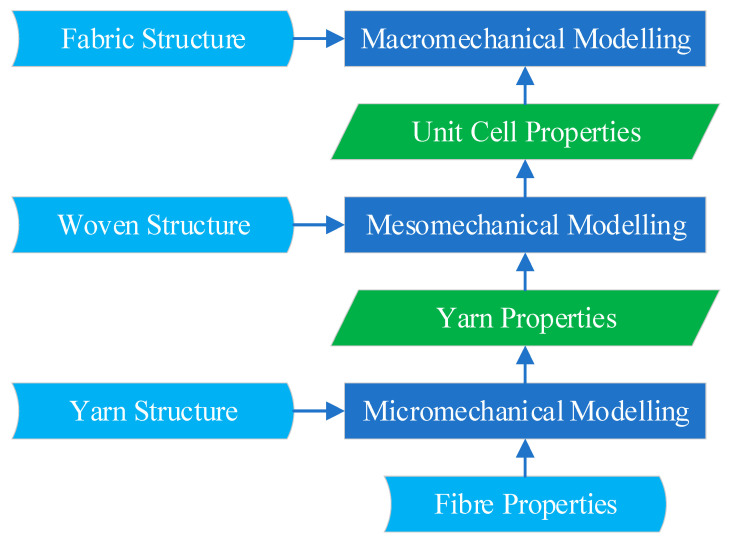
Multiscale analysis hierarchy diagram of woven fabrics.

Any analysis and measurement of fabric property must deal with a large number of fibres and physical parameters spanning multiple scales and orders of magnitude. Choosing to use a macroscopic or microscopic scale to deal with structural and mechanical analyses in which their magnitudes span several orders of magnitude will bring many difficulties and inconveniences. With the assembly of fibres being the main component in a fabric, their presence cannot be ignored, and the fabric cannot be regarded as a single continuous material. Therefore, a hierarchical structure of a multiscale analysis corresponding to the fibre assembly hierarchy emerges, as shown in Figure 1. This multiscale analysis of a fabric structure is divided into three levels: micro-, meso-, and macro-. At the microscale analysis level, the aim is the mapping relationship from fibre properties and yarn structure input to yarn property output. In such hierarchical relationships, yarn properties homogenize yarns from fibre component units into continuous material bodies. At the mesoscale analysis level, yarn properties and woven fabric structural inputs are mapped to fabric unit cell properties. The unit cell properties are homogenized and can be used as input to the next level of processing. Macroscale analysis and modelling levels map fabric unit cell properties and extended fabric sheet structural dimensional inputs into mechanical behaviours such as stretch, shear, and bending of the fabric.

The deformation of fabrics is usually divided into simple deformations such as stretching, shearing, bending, and compression and combinations of complex deformations such as biaxial [1]. For example, in the yarn analysis, when a simple yarn tensile deformation is mapped to the fibre level, what occurs in the fibre is a combined deformation of stretching, bending, torsion, interface friction, and side compression.

In the early days of textile science, the analyses at all levels were based on traditional mathematical and mechanical methods. With the application of digital computers, numerical methods were first used to support discretized calculations of traditional models, and then direct numerical modelling methods were gradually developed with a landmark by Timoshenko and Goodier [2]. Whether it is numerical or traditional analyses, the mapping relationships they describe are consistent. This article therefore does not need to distinguish between them when discussing the internal mapping relationships of fabric mechanics.

The mechanical properties of fabrics are time-dependent, and when fabrics are subjected to repeated loads, there are problems such as the accumulation of plastic deformation and the hardening of fibres [3]. There are also limitations of current low-stress fabric measurement technology because all measurements are performed uniaxially.

In the next section of this paper, the main mechanical models of friction and frictional hysteresis are being reviewed, and related to the mechanical properties of the fabric, and revealing the challenges of the coexistence of the pure elastic and the friction hysteresis components of a fabric. The variability of inter-fibre friction within the fabric is also analyzed, as it is the starting point of the Coulomb friction function. In the third and fourth sections of this paper, the structure of the yarn and its mechanical properties are being reviewed, and the relationship between fabric deformation and stress is being analyzed, respectively. In the last section, this paper discusses and recommends how a unified mathematical reference model may be established for defining the mechanical behaviour of fabrics.

## 2. Friction and Hysteresis Review Model

When two objects experience contact under the influence of a normal force applied to their surfaces, they exhibit a tendency to move in relation to each other. This relative motion gives rise to a force on the contact surface, impeding the movement or the inclination for such movement. This physical occurrence is commonly referred to as friction. It is essential to note that friction is not an inherent property of materials but rather a component property. The force generated by friction is termed as frictional force.

Both static friction and sliding friction act in a direction opposite to the object’s motion or tendency of motion and are tangential to the contact surface [4]. The phenomenon of friction is intricate, and despite ongoing efforts, its complete understanding remains elusive. Current laws and models are derived from empirical observations under specific conditions. Tribology, the study of friction, lubrication, and wear, is an evolving science aimed at comprehending these phenomena.

### 2.1. Coulomb Friction Laws

In 1821, Coulomb consolidated the research findings of Leonardo da Vinci and Amonton, presenting his extensive experiments at the Paris Academy of Sciences [5]. Subsequently, these outcomes were distilled and codified into what is now recognized as Coulomb’s law of friction by later generations [6]. 

Static Friction Law: The maximum static friction force between two contacting objects is directly proportional to the normal pressure applied to the contact surface. This force is influenced by the nature and condition of the contact surface but remains independent of the area and shape of the contact surface. Mathematically, it can be expressed as

(1) $$f_{s}=\mu_{s}N$$

Here, $f_{s}$ represents the static friction force, $\mu_{s}\;$ is the static friction coefficient, and *N* is the normal positive pressure.

Sliding Friction Law: The force of sliding friction is directly proportional to the normal pressure on the contact surface and is not influenced by the area of the contact surface [7]. This relationship is described by the equation

(2) $$f_{k}=\mu_{k}N$$

Here, $f_{k}\;$ denotes the kinetic friction force, and $\mu_{k}$ is the kinetic friction coefficient.

Coulomb’s law of friction lacks a description of the transition process that occurs when objects in contact have a tendency to move toward each other but have not yet experienced significant slip.

### 2.2. The Pre-Slip Model by Dahl

**Figure 2:**
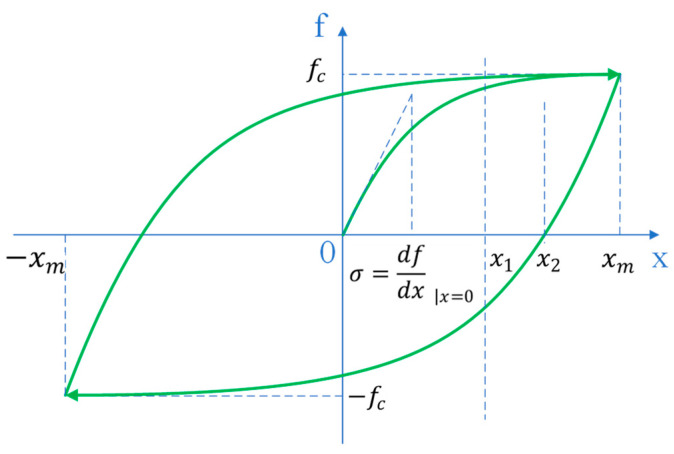
Pre-slip friction model [8], modified by author.

During his experiments, Dahl observed that the relative sliding between the contacting surfaces of objects undergoes a pre-displacement process from a state of rest to sliding [8]. As illustrated in Figure 2, modified by the authors for clarity, when the tangential force on the contact surface is below the maximum static sliding friction force *f_c_*, the object remains relatively stationary on a macroscopic scale. Under the influence of an external force, a gradual and incremental displacement occurs between the contact surfaces of the objects. Upon reaching the point where the tangential force equals *f_c_*, the object transitions into a sliding state. This pre-displacement phase exhibits partial elastic properties. Upon removal of the external force, the objects return a portion of their displacement towards each other but retain some residual displacement. Dahl’s model can be simplified as

(3) $$\frac{df}{dx}=\sigma \left[1-\frac{f}{f_{c}}sgn\left(\dot{x}\right)\right]$$

Dahl’s model employs a first-order system to characterize the pre-slip process of friction. Given that the time increment is always greater than zero in practical operations, *dx* can be employed instead of *x* to ascertain the direction of the sliding. Thus, there exists

(4) $$df=\sigma \left[1-\frac{f}{f_{c}}sgn\left(dx\right)\right]dx$$

In the equation, *σ* represents the friction stiffness, signifying the slope of the friction force f when x=0 and f=0. In Dahl’s model, the interface normal pressure and Coulomb friction remain constants. $f_{c}$ denotes the Coulomb friction force, representing the maximum absolute value of friction force at which the pre-slip process occurs in the model. As *σ* approaches infinity, Dahl’s model converges towards the Coulomb model. Both Equations (3) and (4) have been simplified by the authors.

Dahl’s model functions as an expansion of the Coulomb model, mitigating the uncertainty associated with the Coulomb model when velocity is zero. It replaces the step function with a continuous process described by a first-order differential equation, transforming the friction force from an abrupt jump to a gradual transition along the displacement axis. Importantly, the model unveils the hysteresis relationship of pre-friction. It relies solely on displacement and the sign of the displacement increment, with no connection to velocity, rendering it suitable as a hysteresis operator [9]. the model does not address the inconsistency between static and kinetic friction coefficients, nor does it tackle the modelling challenge presented by the Stribeck effect.

### 2.3. The Friction Model by Bliman and Sorine

Bliman and Sorine expanded Dahl’s friction model in their works [10,11,12]. When employing second-order differentiation, this extension allows for the modelling of static friction and the Stribeck effect. The friction force in the model becomes a function of the path, with no dependence on the speed of the system along that path. In terms of the spatial variable *s*, the representation is

(5) $$s=\int_{0}^{t}\left|v\left(\tau \right)\right|d\tau$$

(6) $$\left\{\begin{array}{c} F=Cx_{s}\\ \frac{dx_{s}}{ds}=Ax_{s}+Bsgn\left(v\right) \end{array}\right.$$

Let $A=-1/\varepsilon_{f}$, $B=f_{1}/\varepsilon_{f}$, and C=1 to obtain the first-order model of Bliman and Sorine:

(7) $$\frac{dF}{dt}=\frac{dF}{ds}\frac{ds}{dt}=\left|v\right|\frac{dF}{ds}=\frac{f_{1}}{\varepsilon_{f}}\left(v-\frac{F}{f_{1}}\left|v\right|\right)=\frac{f_{1}}{\varepsilon_{f}}\left[1-\frac{F}{f_{1}}sgn\left(v\right)\right]v$$

We propose that Equation (7) is the first-order model of Dahl when a = 1. We can obtain a second-order model as a combination of two Dhal’s models, steady-state, and when (*s* → ∞), their difference is the dynamic friction.

### 2.4. Hysteresis and Models

Hysteresis is a phenomenon observed in various fields, including physics, chemistry, biopsychology, and economics. However, there is currently no universally accepted definition for hysteresis. Different disciplines and scholars offer diverse interpretations of the concept. According to Ewing, hysteresis involves periodic changes in parameter x(t), leading to corresponding periodic changes in parameter y(t), forming a hysteresis curve on the (x, y) plane [13]. Mayergoyz defines hysteresis as a multi-branch nonlinear relationship between output and excitation, with a transition occurring between branches at each excitation extremum [14]. Mayergoyz believes that path switching is the essence of hysteresis and looping is its result. There is no one-to-one correspondence between the hysteretic output and its stimulus, and it is not only related to the current incentive, but also to the initial value and the accumulation of past incentive states. The Dahl and the Bliman and Sorine models are two examples of mathematical models used to describe friction-induced hysteresis phenomena.

#### 2.4.1. The Coexistence of Friction Hysteresis and Elastic Behaviour

In the measurement data concerning the low-stress mechanical properties of fabrics, the loading and unloading curves consistently exhibit deviation, creating hysteresis. This hysteresis comprises both purely elastic components and frictional hysteresis components. According to Haas and Dietzius, the friction within the fabric is responsible for the divergence of the loading and unloading curves from the nonlinear elastic curve of the fabric in both positive and negative directions [15]. Conceptually, we can consider the friction hysteresis and elastic forces generated within the fabric as two distinct forces coexisting along the same force action line and force action point. However, their directions and magnitudes follow their respective physical laws.

Even under low-stress conditions, where friction hysteresis undergoes state transitions, the occurrence of slipping phenomena between fibres, leading to fabric disintegration, is unlikely. The mechanical properties of the fabric are influenced and governed by the coexistence of pure elasticity and frictional hysteresis, which jointly dictate fabric deformation. As the degree of fabric deformation varies, the predominant influence of these two coexisting forces on the relative movement between fibres undergoes transitions.

Despite the coexistence of friction hysteresis and elastic forces on the same force action line and point, and sharing the same fabric deformation, they each adhere to distinct regular changes and constitute two independent forces. Although these forces can be synthesized or decomposed through simple algebraic addition and subtraction relationships due to their presence on the same force action line and point, it is crucial to note that both forces are inherently nonlinear. Their relationship represents a combined, nonlinear interaction of forces rather than a linear superposition relationship [1]. Based on the preceding analysis, the stress relationship for fabric deformation under low-stress conditions can be articulated as follows:

(8) $$\left\{\begin{array}{c} \sigma_{x}=S_{1}+H_{1}\\ \sigma_{y}=S_{2}+H_{2}\\ \tau =S_{3}+H_{3}\\ M_{x}=S_{4}+H_{4}\\ M_{y}=S_{5}+H_{5} \end{array}\right.$$

where $\sigma_{x}$, $\sigma_{y}$, τ, $M_{x}$, and $M_{y}$ are the warp/weft tensile stress, shear stress, and warp/weft bending couple, respectively. $S_{i}$ are their corresponding pure elastic components, and $H_{i}$ the corresponding friction hysteresis component.

The simultaneous presence of pure elasticity and frictional hysteresis in fabrics precludes their direct and independent acquisition through instrumental measurement. However, it is recognized that these two components perform distinct roles in dynamical systems and their importance is crucial in defining fabric behaviour. This underscores the significance of employing techniques, such as algorithms (software sensors), to separate and decompose them from measurement data [16].

#### 2.4.2. Fabric Properties’ Equivalent Coulomb Friction as a Function of Deformation

There is a potential for a notable alteration in the equivalent Coulomb friction within the hysteresis component of the fabric’s mechanical properties. Soft fibres, constituting the fundamental elements of fabric structure, are assembled into fabric through repeated curling. Fabric structures rely on the crimping and friction between fibres to interconnect and uphold the physical integrity of the structure. Numerous gaps exist between fibres in the fabric structure. The presence of fibre curls and gaps contributes to the fabric’s “fluffy” appearance, rendering it soft and endowing it with outstanding breathability and thermal insulation properties. The “fluffy” structure of fibres within the fabric creates ample space for fibres to redistribute when subjected to external force, facilitating the easy deformation of the fabric. This characteristic makes the fabric highly malleable after exposure to external forces, contributing to its ability to conform closely to the contours of the human body when used as a clothing material. While these attributes confer excellent wearability, it is during wear that the equivalent Coulomb friction between fibres in the fabric undergoes significant changes due to body movements, stresses, and strains.

In typical mechanical structures, such as the pre-slip process of friction observed on bearings by Dahl, the Coulomb friction force is often considered constant. However, during the tensile deformation of fabrics, the equivalent Coulomb friction force consistently increases with the rise in tensile force and deformation. This phenomenon arises from the self-locking between fibres induced by the twist of the yarn during tensile deformation.

Consider ${f_{c}}_{1}\;$ and ${f_{c}}_{2}$ as the equivalent Coulomb friction forces in the warp and weft directions, respectively. ${f_{c}}_{3}$ represents the shear equivalent Coulomb friction force, while ${f_{c}}_{4}$ and ${f_{c}}_{5}$ denote the equivalent Coulomb friction forces in warp and latitudinal bending. The set of independent variables representing fabric deformation is denoted as ($x_{0},\;x_{1}\ldots x_{n})$. The expression for the equivalent Coulomb friction force of fabric deformation can be formulated as

(9) $$\left\{\begin{array}{c} {f_{c}}_{1}={F_{c}}_{1}\left(x_{0},\;x_{1}\ldots x_{n}\right)\\ {f_{c}}_{2}={F_{c}}_{2}\left(x_{0},\;x_{1}\ldots x_{n}\right)\\ {f_{c}}_{3}={F_{c}}_{3}\left(x_{0},\;x_{1}\ldots x_{n}\right)\\ {f_{c}}_{4}={F_{c}}_{4}\left(x_{0},\;x_{1}\ldots x_{n}\right)\\ {f_{c}}_{5}={F_{c}}_{5}\left(x_{0},\;x_{1}\ldots x_{n}\right) \end{array}\right.$$

In the subsequent article, we will systematically identify the specific independent variables associated with each equivalent Coulomb friction force.

The most important characteristic of hysteresis is its branching behaviour. That is, the current state of hysteresis response is not only related to the current stimulus, but also related to the path of the accumulated memory of past states. The mechanical behaviour of fabrics is irreversible. Since during end use, the extreme points of fabric deformation occur randomly and have infinite possibilities, it is impossible for instrument measurement to traverse creating all extreme points and their branches. Their characterization can only be handled through parametric models. At the same time, the purely elastic and frictional hysteresis components of the fabric’s mechanical properties must be decomposed. This is the second reason why we need to employ techniques such as algorithms (software sensors) to decompose them from measurement data.

Friction hysteresis can be described using the Dahl model of Equation (4) or the Bliman and Sorine Friction Model of Equation (7) when extended. However, it is worth noting that in Section 2, the equivalent Coulomb friction of the fabric mechanical properties has been determined not to be a constant but a multi-variable function. For fabric friction hysteresis, the Coulomb friction constant in Equation (4) or Equation (7) must be expanded into a multivariate function, Equation (9).

## 3. Fibre and Yarn Structure and Their Mechanical Relationship

The objective of a microscale mechanical analysis of fibre components is to investigate the connection between the macroscopic deformation of a yarn and the microscopic response of the individual fibres composing it. With the continuous development of new spinning technologies, there is a growing diversity in the types of yarns that characterize the assembly of fibres. Lawrence, offers a comprehensive introduction to various spinning technologies, the resulting yarns, their structures, fibre distribution relationships, and their physical and mechanical properties [17]. Ognjanovic, conducted an extensive review encompassing analytical, discrete, statistical, neural network, knowledge network, numerical, and fuzzy logic models of yarn [18]. This paper does not aim to replicate these discussions; instead, it will concentrate on reviewing specific aspects of microanalyses and modelling related to friction in fibres and yarns.

### 3.1. Fibres

A fibre constitutes the fundamental unit of a fibrous material structure and serves as the primary locus of friction within the material. While friction predominantly occurs on the fibre surface, it is influenced not only by the surface morphology but also by the overall properties of the fibre. Research indicates that factors such as surface morphology, cross-sectional size, shape, and mechanical properties—including shear, tensile, flexural modulus, and transverse compression properties—are interconnected with the frictional characteristics of the fibre. Environmental conditions, such as temperature and humidity, along with the use of fibre surface conditioners, also impact the fibre’s frictional behaviour.

Polymer fibres exhibit a two-phase structure comprising distinct crystalline and amorphous regions with well-defined boundaries. Loading and unloading curves for fibres with such a structure often exhibit hysteresis, displaying time-dependent behaviours and energy loss. These fibres, known as viscoelastic fibres, demonstrate dimensional creep and stress relaxation.

The relationship between the friction coefficient among polymer fibres and their relative sliding speed is complex and nonlinear. The friction coefficient and relative slip velocity dependence curve reveals a concave point from zero slip to the maximum slip velocity. The presence of scales in the surface morphology of many natural protein fibres contributes to anisotropic friction coefficients between fibres. Additionally, the disparity between static and dynamic friction coefficients results in stick–slip phenomena when the relative sliding speed of friction points between fibres is less than 0.1 m/min [19]. 

Therefore, we know that the friction and normal pressure of polymer fibres follow a power law:

(10) $$F_{f}=aN^{n}$$

Here, $F_{f}$ represents the friction force, *N* represents the normal force, and *a* and *n* are regression constants derived through data fitting, with n < 1, and approximately equal to 2/3.

Friction between fibres is linked to the initial stress on the surface. Heat setting in finishing can significantly impact the friction behaviour between fibres by largely eliminating these stresses and resetting the stress distribution state between fibres. Different materials exhibit varied heat-setting principles and specific temperatures, leading to diverse heat-setting effects. This process can be utilized to adjust the internal friction of fabrics, and comparing the directional differences in mechanical properties before and after heat setting can provide valuable insights of its behaviour [20]. 

### 3.2. Yarn Structure

Yarn geometry is the basis for a yarn mechanical analysis. The morphology of fibres in yarn is affected by many factors such as spinning technology as well as fibre type and properties. Different spinning methods mean different fibre assembly relationships and yarn structures.

The fibres in the fibre bundle become parallel and straight to each other after processing such as carding. After twisting and winding, the fibres form a spiral structure along the axis of the yarn. The early yarn structure model established by Hearle simplified the helical structural components of the fibres in the yarn into a surreal and simple spiral [21]. The pitch and radius of the helix will remain unchanged. The fibres spiral around the yarn spool, forming layers. The fibre at the centre of the yarn can be thought of as a spiral with zero radius. They further described the geometry of the helical structure, in which the elastic fibres follow a perfect spiral path and are evenly distributed over the yarn cross-section. These simple spirals that ignore the randomness and complexity of yarn components fail to describe real yarn structures, but they provide a starting point for modelling them.

Morton and Yen, traced fibres using colour contrast to track the actual position and direction of fibres in a yarn [22]. They sampled yarn with tracer fibres and immersed it in a methyl salicylate dispersion; the sample had excellent transparency in liquids. The yarn sample and the tracer fibres within it were projected onto a plane through a microscope. Repeating damped sine waves could be observed in the projection plane, and fibres moving back and forth between the core and surface of the yarn. This phenomenon is called “fibre migration”. It shows that the helical radius of the fibre in the yarn changes back and forth between the core and the surface of the yarn along the yarn axis with some statistical regularity. Hence, fibres are intertwined with repeated migrations in the 3D space of the yarn. The cause of fibre migration is attributed to an imbalance in the tension on the fibres from the inside to the outside of the yarn. After this, many papers on fibre migration research were published, and more causes and mechanisms leading to fibre migration have been explored [23,24,25,26]. 

Nowadays, the geometric structure model of the yarn can be generated with the help of computer software, and the three-dimensional effects of fibres and yarns can be displayed through data visualization methods [27,28,29]. 

Zhao et. al. used micro-CT scanning technology to obtain the actual fibre position and direction of real yarns and fitted them to the yarn geometric model [30]. The fitting has taken into account the statistical properties of the fibre, factors such as yarn twist, fibre migration, fibre deformation, and surface hairiness. In consequence, the digital geometric model of the yarn can be very close to the real yarn structure.

#### 3.2.1. Yarn Stretching

The fibres used to assemble yarn can be divided into filaments and staple fibres according to their length. A filament is usually made by man-made fibre processing, and a natural silk filament can also be obtained through a reeling process. Most natural fibres such as cotton and wool are staple fibres. Man-made fibre filaments can also be processed into staple fibres through pulling and cutting processes. The principles of tension transfer are significantly different between yarns made of long filaments and yarns made of staple fibres. Filament yarns formed by twisting a multifilament can directly transmit tensile forces in the length direction. For staple fibre yarns, due to the entanglement between fibres caused by fibre migration, the yarn can transmit tensile force at any required length through the frictional self-locking relationship between fibres formed after twisting. Whether a filament or staple fibre yarn, friction and twisting between fibres can maintain the physical structural integrity of the yarn.

Gegauff [31], when analyzing the strain relationship between filament fibres and the yarn structure they constitute, concluded that the relationship between fibre elongation $\varepsilon_{f}$, yarn elongation $\varepsilon_{y}$, and twist angle θ is

(11) $$\varepsilon_{f}=\varepsilon_{y}{cos}^{2}\theta$$

Platt suggested that the yarn modulus decreases with increasing twist and that this decrease is related to the stress distribution due to yarn geometry [32]. This conclusion is consistent with the model of Gegauff [31]. Hearle, gives a set of equations and a detailed derivation analysis for the load–extension behaviours of twisted yarns [33]. The system of equations expresses the load–extension relationship for twisted filament yarns. They can be used to predict stress, shrinkage, twist angle, and Poisson’s ratio in yarns. Based on a simplified fibre helical structure, Hearle created the first yarn continuity model [21]. Huang and Funk [34] used the elastic bending rod theory to study the small elongation of the yarn, improving the previous model of Hearle, and their model was compared with the models of Gegauff [31] and Hearle [21] and verified with measured data. Van Langenhove, created an FEA yarn model that allowed fibre slippage in the yarn. The model involves inter-fibre friction and considers stress changes in the fibres as a function of fibre length [35].

Elmogahzy, reviewed the relationship between yarn strength and twist and inter-fibre friction [36]. The concept of “best twist” is introduced. Untwisted fibre strips have only weak interfacial friction bonding force. As the twist increases, the normal force on the fibre surface increases, and the interface friction between fibres in the yarn increases. More cross-linking points between fibres reach or exceed frictional self-locking conditions, increasing yarn strength. After reaching a critical point, a further increase in twist will cause excessive tilt between the fibre and the yarn axis, and the fibre will fail due to excessive stress. This critical point is the “optimal twist” of the yarn. The increase in the fibre interface friction coefficient will allow the yarn to have enough fibre cross-linking points to reach or exceed the friction self-locking condition and achieve the optimal twist under a smaller twist, thereby improving the strength of the yarn. It is worth noting that changing the twist has many effects on the yarn. While changing the strength of the yarn, the increase in twist also results in a decrease in the bulk or softness of the yarn, which changes the feel and warmth retention ability of the yarn. Therefore, the twist that is optimal for yarn strength may not be optimal for the overall performance of the yarn.

#### 3.2.2. Yarn Bending

The fibres are bent and twisted in the yarn. Backer, by analyzing the geometric structure of the yarn, explored the fine-tuning of the local position of the fibre within the yarn and the corresponding stress relationship when the yarn was bent [37]. The existence of friction between fibres constrains the length transfer and micro-motion between fibres, resulting in changes in stress within the fibres. Due to the existence of twisted helices, the tensile stress on the fibre helices outside the bend leads to an increase in the lateral yarn pressure component. The increase in fibre surface pressure in turn leads to an increase in the frictional resistance between fibres. Grosberg also analyzed the assumed interaction between the fibre layers when analyzing the bending behaviours of the fabric, in particular the relationship between the frictional bending resistance couple and fibre slippage [38]. The model involves the normal pressure between fibres, the friction coefficient between fibres, and the external equilibrium couple. He believed that the normal pressure between fibres was formed during processing and did not involve changes in the normal pressure between fibres that may be caused by combined forces such as stretching of the fabric. The fibres in his model are in two states, slip and non-slip, and the transition process of fibres between non-slip and slip is not explored. Park and Oh investigated the bending stiffness of the yarn by two methods: a theoretical analysis related to the microstructure of the fibres composing the yarn and an experimental analysis related to the geometric deformation [39]. Their research shows that as the twist of the yarn increases, the bending stiffness of the yarn decreases. Increasing the ratio of the shear modulus to the tensile modulus of the fibre increases the bending stiffness of the yarn. Bral applied micro-computed tomography imaging technology to extract the geometric model of the physical yarn. Finite element simulation is then used to simulate the bending and stretching behaviour of the yarn. Their research showed that reducing the twist in a yarn increases its bending stiffness [40]. However, changing the friction coefficient between fibres has shown little effect on the bending stiffness of the yarn, which may be the result of the initial condition settings of the simulation. The state can be changed when the yarn is stretched, causing it to become compacted and create a large number of fibre bonding points. The twist of the yarn has a significant impact on the tensile properties and bending stiffness of the yarn under the combined stress state of the yarn being stretched.

### 3.3. Discussion Points on Yarn Friction

Due to the entangled fibres formed by twisting and fibre migration, the fibres form a self-locking relationship through the friction points between them, and there is tension transmission at any yarn length. Moreover, the Coulomb friction force formed between fibres increases with the increase in tensile force, creating a unique stretch hysteresis in the fabric. The yarn twist and the interfacial friction capacity of the fibre will significantly affect the hysteresis behaviour of the yarn. Yarn twist also significantly affects the yarn’s ability to stretch.

From the perspective of tensile strength, there is an optimal twist for staple fibre yarns. When the twist is too low, the yarn will disintegrate due to insufficient radial centripetal force (equivalent friction self-locking angle) when stretched. When the twist is too high, the yarn will fail due to excessive tensile stress within the fibre.

The interfacial friction capacity of fibres has a significant impact on the tensile strength of yarns. When the surface friction coefficient of the fibre increases, the optimal tensile strength twist of the yarn decreases, the tensile stress on the outer fibre is reduced, and the tensile strength of the yarn is increased.

The bending section modulus of the yarn decreases with increasing twist when not stretched. When the combined deformation of stretching and bending occurs, if there is appropriate twist, it can be speculated that on the one hand, the presence of the spiral component reduces the bending section modulus of the yarn. On the other hand, when the yarn is stretched, the tensile force on the yarn is decomposed into the tensile force and the radial cohesion force of the fibre. The radial cohesion force of fibres will increase the Coulomb friction force between fibres and enhance the bending resistance section modulus of the yarn. The higher the tensile force on the yarn, the greater its resistance to bending. The higher the surface friction coefficient between fibres, the higher the ratio of the increment of the yarn’s bending section modulus to the increment of the tensile force exerted on the yarn. Therefore, under compound deformation conditions, for filament yarns, when the twist of the yarn increases near the zero point, its bending modulus should increase. However, a further increase in twist will lead to a decrease in the bending modulus of the yarn. For staple fibre yarns, since the minimum twist must ensure the self-locking relationship between fibres, a further increase in twist will inevitably lead to a decrease in the bending modulus of the yarn.

## 4. Fabric Deformation and Stress

The deformation of woven fabrics can be classified into in-plane deformation and bending deformation. In-plane deformation can be further divided into tensile deformation and shear deformation.

### 4.1. In-Plane Deformation of Woven Fabrics

The plane deformation of woven fabrics includes tensile and shear deformations. Depending on the direction of the shear angle, shear deformation can work in the positive and negative directions. Because the fabric is a soft sheet material, when the fabric is subjected to a compressive force opposite to the tensile direction, it quickly becomes unstable and enters a buckling state. The analysis of the buckling state is beyond the scope of plane deformation, so the plane deformation of fabric only provides a tensile and shear deformation analysis.

#### 4.1.1. Tensile

Woven fabrics are usually made of interwoven warp and weft yarns. Before being deformed by tensile force, the internal stress of the fabric after shaping is in the lowest stable state. The warp and weft yarns of the fabric are in a mutually orthogonal geometric relationship. The tensile mechanical behaviour of the fabric discussed in this section refers to the stress–strain relationship of the fabric when the fabric is stretched along the warp and weft directions separately or simultaneously.

In Section 2 we have made it clear that the pure elastic and the friction hysteresis component in the mechanical properties of fabrics always coexist. However, for the convenience of modelling, we still discuss them separately as much as possible.

#### 4.1.2. Fabric Low-Stress Tensile Deformation and Crimp Exchange between Warp and Weft Yarns

Before tensile deformation occurs, the yarns inside the shaped fabric are in a sinusoidal crimping state. Since the yarn itself may have twist, the fibres in the yarn are in a more complex compound crimp state. When tensile deformation begins to occur, from a mesoscopic perspective, the change in the crimp state of the yarn dominates the stress–strain behaviour in the initial stretching stage.

As the x-direction yarn begins to stretch, its crimp is gradually straightened. And pressure is formed at the intersection with the y-direction yarn. Assuming that the macroscale tensile displacement of the y-direction yarn is zero, the y-direction yarn is further bent under the action of pressure at the intersection with the x-direction yarn. And because the yarn is bent, the yarn in the y-direction is also stretched in the fabric. The deformation of y-direction yarns inside the fabric results in the formation of bending stress and tensile stress in the y-direction yarns. Whether the x-direction yarn is straightened and the x/y-direction yarn is compressed laterally, or the y-direction yarn is further bent and internally elongated, they only need to exert a small tensile force on the yarn in the x-direction. In the initial stretching stage when the crimp deformation of the yarn dominates the deformation of the fabric, the initial tensile modulus of the fabric is often very low.

From a microscopic scale, the change in the fibre crimp state also dominates the stress–strain behaviour of the yarn in the initial stretching stage. At the beginning of the initial stretch, the yarn is in a “fluffy” state in both directions. When subjected to tension, the fibres in the yarn are gradually tightened. In the x-direction yarn, the cross-section of the yarn becomes more rounded due to the centripetal tightening force generated by the twist on the fibre bundle [33]. As tension increases, the fibres in the yarn are held tight in both directions. The friction between them increases, resulting in a gradual increase in the bending stiffness of the yarn.

When the woven fabric is stretched along the warp direction, the warp yarns are gradually straightened, and the crimp is reduced. This causes a gradual increase in weft crimp. Or when the weft yarn is gradually straightened, this causes the warp yarn to increase in crimp. This is known as crimp interchange. Accompanying the crimp interchange of yarn is the exchange of part of the energy between them. The crimp interchange between yarns is achieved by a dynamic balance of pressure at the interlacing locations of the yarns [41]. Another paper by Kawabata [42,43,44] also describes this balancing process in an analytical model.

**Figure 3:**
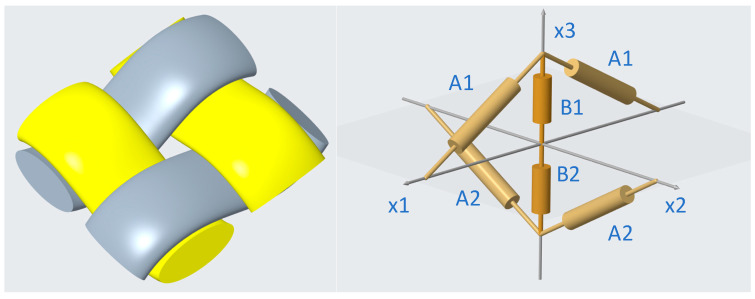
Schematic diagram of simplified unit cell model of fabric from [42].

Kawabata constructed a biaxial tensile analytical model for plain weave fabrics at the mesoscopic scale [42]. The model describes the relationship between warp and weft yarns using the three-dimensional geometry shown in Figure 3 It maps the stress and deformation of the fabric unit cell into the stress and deformation of the yarn. In the model, the A1 and A2 distributions represent the nonlinear relationship between tensile stress and strain of warp and weft yarns, and B1 and B2 represent the transverse nonlinear compression characteristics of warp and weft yarns, respectively. In the initial state, the origin of the plane reference coordinates coincides with the intersection of the warp and weft yarns. The axis (x1) is the warp direction, the axis (x2) is the weft direction, and the B1 and B2 points after the warp and weft are bent and located on the axis (x3). The model assumes that the warp and weft yarns are perfectly flexible. Forces due to fibre and yarn bending are ignored.

When the unit cell is subjected to balanced stretching in the positive and negative directions along the x1 and x2 axes, the warp and weft yarns are stretched. But their contact points remain coincident with the origins of x1 and x2. Corresponding pressure is generated at the contact point of the two yarns and causes crimp exchange between them.

Due to the existence of the crimp exchange phenomenon, the elongation in the warp and weft directions contributes close to the same order of magnitude to the stress amplitude in the warp direction. Based on the proportional relationship between different elongations in the warp and weft directions, Kawabata’s calculation results of the theoretical model and the experimental data curve prove this point [42]. Similarly, the elongation in the warp and weft directions also contributes to the stress amplitude of the weft yarn close to the same order of magnitude.

The above simple unit cell model of plain weave fabric shows that the elongation in the warp direction of the fabric is the main factor in forming the stress of the warp yarns. The elongation in the weft direction of the fabric results in changes in weft yarn stress. By crimp exchange, it can have an effect on warp stress that is close to the same order of magnitude. The weft elongation of the fabric is also an important factor affecting the warp stress. The large deformation characteristics of the fabric cause the shear deformation to significantly change the orthogonal relationship between x1 and x2 and the transverse compression performance of the warp and weft yarns. It also changes the crimp exchange relationship and is affecting warp stress.

When the x-direction and y-direction yarns are stretched simultaneously at a ratio of 1:1, the stretching process is similar to when the stretching displacement of the fixed y-direction yarn is zero and only the x-direction yarn is stretched. However, since the weft direction is stretched simultaneously, the elastic modulus of the entire stretching process will be higher than when the tensile displacement of the yarn in the y-direction is fixed to zero. Friction within the fabric will also consume more energy.

When stretching in the x-direction, and the stress in the y-direction is kept at zero, negative macroscale lateral strain is an important feature of this stretching scenario, which is nothing other than classic uniaxial stretching. The entire stretching process is similar to the tensile loading and unloading process that occurs when the tensile displacement of the fixed y-direction yarn is zero. However, since the stretching force of the yarn in the y-direction is zero, when the yarn in the x-direction is stretched, the yarn in the y-direction is further bent, and the fabric shrinks in the y-direction. Hence, the strain of the projected length of the yarn on the fabric plane in the y-direction is negative. Since the yarn loses tension in the y-direction, the elastic modulus of the entire stretching process will be lower than that of the first two scenarios. Friction within the fabric will also consume less energy.

Since the effects of yarn bending stiffness and friction are ignored in [42], this model cannot be used to explain the physical phenomena observed in uniaxial stretching, a special case of biaxial stretching. Kawabata extended further the biaxial tension model [43]. Uniaxial stretching means that when the fabric is stretched along one of its yarn axis directions, the tensile force on the yarn in the other yarn axis direction orthogonal to it is zero. As the tensile force in the stretching direction increases, the yarn is gradually straightened. The yarns in the direction orthogonal to it are further bent, and the overall size of the fabric in this direction is compressed and reduced, a phenomenon defined by Poisson’s ratio.

Assume that the fabric is stretched in the x1 direction, and the strain produced is $\varepsilon_{1}$. Correspondingly, when the fabric is compressed in the x2 direction, the strain produced is $\varepsilon_{2}$; then,

(12) $$\nu =-\frac{\varepsilon_{2}}{\varepsilon_{1}}$$

v is the Poisson’s ratio of the fabric. It provides partial information for characterizing the tensile mechanical relationship properties of the fabric in its two yarn axes. However, it is worth noting that the fabric is an anisotropic nonlinear structure, and the physical meaning of its Poisson’s ratio is different from that of the Hooke body material. In addition, there is also a complex nonlinear functional relationship between the Poisson’s ratio of the fabric and $\varepsilon_{1}$.

The phenomenon that the yarns in the direction orthogonal to the stretching axis are further bent and cause the transverse dimensions of the fabric to be compressed does not only occur in uniaxial stretching. At the intersection of two yarns, when the pressure generated by stretching the transverse yarn is not high enough to balance the increased pressure caused by stretching the longitudinal yarn, further bending of the transverse yarn can occur. As the tensile force in the x-direction further increases, the initial stretching state dominated by the “fluffy” state and crimp state of the yarn gradually transitions to the post-stretching state. In this state, the elongation of the fabric is mainly contributed by the elongation of the yarns. The tensile modulus gradually transitions to nearly linear and is much larger than the initial modulus. After the tensile deformation reaches the extreme point, the fabric enters the unloading recovery process. During the unloading process, the elastic potential energy accumulated in the warp and weft yarns is gradually released. Since internal friction consumes part of the energy, residual deformation and residual stress will be left in the fabric when the overall elastic potential energy is exhausted.

#### 4.1.3. Effect of Shear Deformation on Tensile Stress–Strain Relationship

Kashani studied the tension–shear coupling behaviour of woven fabrics. Also included is the effect of fabric shearing on yarn stretch. Their research shows that the coupling goes both ways [45]. That is, the tension of the fabric affects its shear behaviour, and conversely, the shear deformation also affects the stretching of the fabric. They observed in their tests that fabric shear reduced its tensile stiffness.

Launay used their picture frame shear test device that can measure simultaneous tension to observe the coupling of shear and tension. They observed that when the fabric was kept in the tensile direction without elongation, the tensile stress in both yarn directions of the fabric changed significantly as the fabric was sheared during the test [46]. Komeili and Milani, also observed in their experiments that biaxial tension changes in the fabric yarns were induced when the fabric was sheared [47].

When the tensile displacement of the yarns in the x- and y-directions is fixed to zero and the fabric is sheared and deformed under force, corresponding stresses are induced in the yarns in the x- and y-directions. The effect of shear on stretching also alters the orthogonal relationship between the x and y yarn directions [48]. For the same external force, it is equivalent to changing the stretching direction, which may require further angle projection processing and have different stress–strain relationships.

#### 4.1.4. Effect of Bending Deformation on Tensile Stress–Strain Relationship

In the above analysis process of the stress–strain relationship of the fabric’s in-plane deformation, we did not include the influence of the fabric’s bending deformation. We have not found enough evidence or information about this. However, in the interaction between fabrics and fluids such as air, there are indeed scenes of combined deformation of bending, stretching, and shearing. The change in the fibre spatial distribution relationship caused by bending will inevitably affect the tensile and shear properties. However, under normal conditions, the stress amplitude due to bending deformation is much smaller than the stress amplitude of tensile and shear deformations.

#### 4.1.5. Stress–Strain Relationship of Tensile Elastic Deformation

To sum up, the pure elastic components *S*_1_ and *S*_2_ of the fabric’s warp and weft stretch in Equation (8) can be expressed as nonlinear functions of the warp deformation $\varepsilon_{x}$, weft deformation $\varepsilon_{y}$, and shear deformation γ of the fabric. That is,

(13) $$\left\{\begin{array}{c} \sigma_{x}=S_{1}\left(\varepsilon_{x},\;\varepsilon_{y},\;\gamma \right)+H_{1}\\ \sigma_{y}=S_{2}\left(\varepsilon_{x},\;\varepsilon_{y},\;\gamma \right)+H_{2} \end{array}\right.$$

If the influence of longitudinal and latitudinal bending deformations $K_{x}$ and $K_{y}$ is not ignored, then

(14) $$\left\{\begin{array}{c} \sigma_{x}=S_{1}\left(\varepsilon_{x},\;\varepsilon_{y},\;\gamma ,K_{x},K_{y}\right)+H_{1}\\ \sigma_{y}=S_{2}\left(\varepsilon_{x},\;\varepsilon_{y},\;\gamma ,K_{x},K_{y}\right)+H_{2} \end{array}\right.$$

#### 4.1.6. Inter-Fibre Friction during Tensile Deformation

Intra-fabric friction caused by fabric stretching has a unique behaviour. During the initial stretching phase, the contact points between fibres are redistributed. Frictional self-locking occurs between fibres due to the twist of the yarn and the intertwining of the fibres. As the tension increases, the contact points between fibres and the normal pressure at the contact points also increase. This leads to an expected increase in Coulomb friction. Although the actual friction force is constantly approaching the Coulomb friction force, friction is always in a transition process. In addition, the transverse compression of the yarns caused by the increasing pressure between the x- and y-direction at the yarn interlacing points also contributes to the increase in internal friction.

As the tensile force further increases, the initial stretching state dominated by the bulky and crimped states of the yarn gradually transitions to the post-stretching stage. At this stage, due to the self-locking of the yarn, the normal pressure at the contact point between the fibres always increases with the increase in the tensile force. The expected value of Coulomb friction for the internal friction of the fabric also increases. Internal friction has also been in the process of transition. After the tensile deformation reaches the extreme point, the fabric enters the unloading recovery process. Since the direction of tensile deformation is reversed at the extreme point, the Coulomb friction force is reversed. Branch formation hysteresis occurs due to friction within the fabric, and friction drops off sharply along new branches. If the unloading deformation process is switched to the loading deformation process again, the friction within the fabric will branch again.

To sum up, we can conclude that due to the frictional self-locking relationship, the Coulomb friction of the woven fabric is directly related to the tensile force. The independent variable of all tensile forces is also the independent variable of its Coulomb friction.

### 4.2. Shear

Further work on fabric shear modelling was conducted by Kawabata [44]. In this shear model, the positive pressure of the yarns at the intersection point is determined by the strains of the warp and weft yarns. They are important factors in forming the shear torque between yarns. They are also important factors in determining shear hysteresis. Due to homology, shear hysteresis is correlated with shear stiffness. A Kawabata biaxial tensile tester was used for shear measurement experiments. Similar to the way the commercial KES test system tests fabric shear, the test instrument does not have the ability to directly control the transverse yarns. That is, the instrument does not have the ability to control the positive pressure of the yarn at the intersection point in the test.

Measurement of tension during picture frame shear testing shows that different settings of warp and weft tension significantly change the shear response [46]. As the tension increases, the shear force increases. Kashani observed the same results in experiments investigating the tension–shear coupling behaviour of woven fabrics [45]. Wang constructed a tension–shear coupling analysis model that can explain the large shear deformation of woven fabrics. An experimental device that can simultaneously measure fabric biaxial tension and shear was used to test fabric samples to verify the validity of the model. Its validation experiments show that the effect of tension on shear properties is directly reflected in the energy dissipation of shear friction. Increased tension results in increased contact forces at yarn intersections, thereby increasing the shear friction couple. Tests have shown that the shear stiffness of the fabric increases significantly with increasing tensile force due to higher friction resulting from higher tension [49]. Crossing and overlapping of yarns are important sources of tension–shear coupling.

#### Shear Deformation

The fabric shear couple mainly consists of two parts. That is, the friction couple formed by the intersection of yarns and the force couple formed by lateral extension between yarns. In the initial stage of shearing, the force on the unit cell is smaller than the Coulomb friction couple at the intersection of the yarns. In the transition process of friction hysteresis, dominated by elasticity, the yarn is gradually twisted and undergoes small angle deflection. When the force on the unit cell gradually increases to be greater than the Coulomb friction couple, the crossed yarns begin to rotate around the centre of the cross. The area of yarn interlacing gradually increases. When the shear deformation of the woven fabric increases to the point where structural locking occurs, adjacent yarns contact and squeeze each other; the onset of jamming becomes the main contributor to the shear resistance. The shearing of clothing fabrics generally tends to buckle out of their plane when reaching the structural locking stage, but this does not happen often. The large macroscopic deformation characteristics of fabric will affect its shear and other mechanical properties [50]. For the shear deformation of fabrics used in clothing, since the maximum shear angle is relatively limited, shear usually does not reach the structural locking stage, jamming, and hence yarn jamming is not the main source of the shear couple. Therefore, the equilibrium relationship of the pure elastic component of shear and the equilibrium relationship of the hysteresis component are highly homologous, and they usually exhibit a high degree of correlation.

Once the shear deformation of the fabric occurs, the orthogonal relationship between the warp and weft yarns of the fabric is changed. The anisotropic fabric model is often based on the orthogonal relationship between warp and weft yarns. However, the orthogonal relationship between warp and weft yarns only exists in zero-shear deformation, and their universal relationship should be non-orthogonal. This causes changes in the stretching and bending relationships of the fabric along the warp and weft directions; hence, fabric mechanics models become more complex.

For clothing fabrics, since the shear deformation is limited, for example, the maximum shear angle is only observed to be within seven degrees, and the error caused by approximating the orthogonal relationship may be acceptable. It is a compromise between computational error and computational convenience.

**Figure 4:**
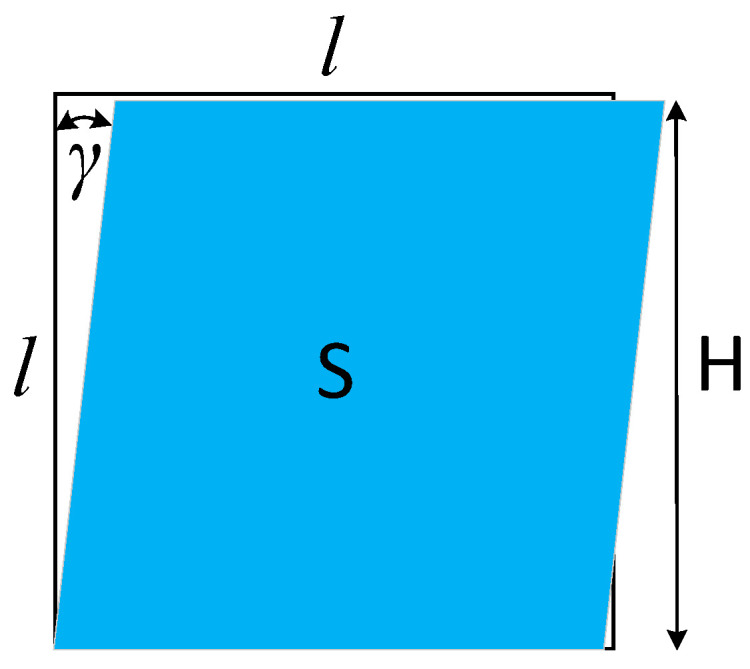
A schematic diagram of fabric shear deformation.

When shearing occurs, the fibre distribution of the fabric changes significantly. Taking photo frame shearing as an example, before the shear deformation occurs, the photo frame is rectangular, and the plane area S of the fabric enclosed by the photo frame reaches its maximum value. As shearing occurs, shown in Figure 4, the area enclosed by the photo frame becomes smaller as the shearing angle increases. The relationship between the area S enclosed by the photo frame and the shear angle γ is

(15) $$S=l^{2}cos\gamma$$

The distance H between the two pairs of parallel sides l of the photo frame also gradually becomes smaller:

(16) H=lcosγ

Shear deformation causes the area of the fabric enclosed by the picture frame to decrease with an increasing shear angle, meaning that the density of fibres within the fabric gradually increases.

### 4.3. Bending

The bending of fabric is one of its important mechanical properties. It enables fabrics to complete 2D/3D transformations in end use. Garments can fit the curved surface of the human body with ease, comfort, and beauty. But the current understanding of fabric bending is still insufficient. Grosberg also believed that the bending behaviour of cloth is divided into a nonlinear part caused by friction and an elastic part. He points out that they originate from yarn resistance in the bending direction, unspecified interactions between yarns, and frictional constraints [38]. The literature on the coupling relationship between bending deformation and other fabric deformations is very limited. Fabrics usually have very small thickness compared to length and width dimensions. They also have a very low flexural section modulus and are very sensitive to the bending moments they experience. This makes fabric bending very susceptible to other fabric deformations while it has a limited effect on other fabric mechanical properties. The drape properties of a fabric are also more sensitive to its bending properties than to its tensile and shear properties.

Shearing of the fabric changes its fibre density and the directional relationship of the warp and weft yarns. Changes in these relationships will inevitably cause changes in the bending section modulus of the fabric. Hu introduced the dependence of fabric bending properties on its yarn orientation. The effect of fabric shear on its bending properties was demonstrated [3]. We have discussed the combined deformation of yarn stretching and bending in Section 3.2.1 and Section 3.2.2 of this paper. The tensile force of a yarn can significantly affect its bending stiffness. The tensile deformation of the fabric also changes the distance between axially parallel yarns and the extension relationship between adjacent yarns during bending. Tensile and shear deformation also affects the friction point distribution between fibres and the positive pressure on the fibre surface at the friction points, thereby changing the bending friction hysteresis. Fabric bending is a complex stress–strain relationship. Under current measurement technology conditions, there are still huge difficulties and challenges in how to obtain the necessary measurement data to verify these hypotheses. And we estimate that with the current rate of progress in this field, these difficulties and challenges will be difficult to solve in the short term.

### 4.4. Other Studies on Fabric Friction

Although friction is recognized as a crucial aspect of fabric behaviour, most recent investigations fall short of addressing friction hysteresis in relation to low-force mechanics. For example, Jeddi studied the influence of fabric structure on the frictional characteristics of woven fabrics, examining the relationship between the fabric-structural-asperity index (FSAI) and frictional parameters. While the results suggest that FSAI is useful for explaining the frictional behaviour of woven fabrics, the study does not relate this to fabric mechanics or provide a fundamental understanding of generic friction occurrences [51]. Hong and Jyaraman addressed the frictional behaviour of fibrous assemblies, emphasizing how fibre friction, influenced by surface structure and material properties, impacts yarns, ropes, and fabrics. They discussed key frictional parameters such as static and kinetic coefficients, frictional force, and friction indices. The paper also covers factors influencing fibre friction, empirical models, and measurement methods for fibres, fabrics, ropes, and geotextiles, but it does not adequately address fabric mechanics [52]. Huang provided a numerical analysis of the effects of inter-yarn friction on the responses of woven fabrics with different weaves (plain weave, 2/2 twill, 2/2 basket, and 3/1 twill) to low-velocity impact [53]. Like many recent publications, this study does not reference fabric mechanics or friction hysteresis behaviour.

### 4.5. Discussion Points on Fabric Friction

From the above considerations, it is inevitable that there are similarities between tension, shear, and bending and that perhaps a unified mathematical reference model may be possible. Bending and shear have obvious similarities, and after normalizing them, we may be able to process their different mechanical properties in the same way. Tensile measurements differ from shear and bending characteristics, as their data curve is confined to the first quadrant of the coordinates. To create a unified mathematical model for tension, we can mirror the stress–strain relationship of $\varepsilon_{x}$ or $\varepsilon_{y}$ in the positive direction of their coordinate axes to the negative direction with the zero point as the symmetry point. Calculating the imaginary part is unnecessary, of course. Furthermore, all independent variables in the fabric mechanics model are real. Through normalization, their definition interval on respective coordinate axes is the closed interval [−1.0, 1.0]. Disregarding the specific physical implications of tensile, shear, and bending properties, a normalized unified mathematical reference model can be abstracted, in which the Dahl’s model can be used to describe friction hysteresis, or the Bliman and Sorine second-order model can be used instead of the Dahl model. The Weierstrass’s first function approximation theorem [54] can then be used to approximate continuous functions. The pure elastic components and Coulomb friction function for the tensile, shear, and bending mechanical properties in the fabric mechanical model can be represented by corresponding one-variable or multi-variable polynomials. Fabrics exhibit different levels of nonlinearity, as observed from the authors’ experience in processing uniaxial measurement data. Utilizing software for the decomposition and characterization of pure elastic and friction hysteresis components in fabric measurement data can serve to validate the rationality and effectiveness of models and algorithms, based on the regularity of fabric friction hysteresis.

## 5. Knitted Fabrics

Understanding friction hysteresis behaviour in woven and knitted fabrics involves examining how these two types of fabrics respond to cyclic loading and unloading. Despite structural differences between woven and knitted fabrics, they share the same fundamental principles.

Both woven and knitted fabrics experience friction hysteresis, which involves energy dissipation during cyclic loading and unloading. This principle is consistent across different fabric structures, and for both types, factors such as fibre type, yarn structure, and the presence of finishes or coatings influence friction hysteresis behaviour. However, the structural characteristics of woven and knitted fabrics lead to distinct differences in their behaviour. Woven fabrics are made by interlacing two sets of yarns (warp and weft) at right angles, creating a more rigid and stable structure. This structure leads to less deformation under load, resulting in lower friction hysteresis. In contrast, knitted fabrics are made by interlooping yarns, resulting in a more flexible and elastic fabric. This structure allows for greater deformation under load, leading to higher friction hysteresis due to more significant energy dissipation during cyclic loading. The differences in deformation and recovery further highlight the contrast between woven and knitted fabrics. Woven fabrics typically show less deformation and quicker recovery after loading due to their tight and stable structure, resulting in relatively low energy loss due to hysteresis. Knitted fabrics, on the other hand, exhibit greater deformation and slower recovery after loading because of their looped structure, leading to higher energy loss and greater friction hysteresis.

When the knitted structure is more elastic and has good recovery, however, the friction hysteresis behaviour of knitted fabrics shows some variations compared to less elastic and slower recovering knitted fabrics. This higher elasticity and better recovery impact the energy dissipation and deformation characteristics under cyclic loading and unloading, showing more efficient recovery after deformation, which modifies the hysteresis behaviour slightly. Elasticity and energy dissipation in highly elastic and well-recovering knitted fabrics mean that they can stretch significantly under a load but also return to their original shape more efficiently. This improved recovery reduces the amount of energy lost in the form of heat during the hysteresis cycle. As a result, while the friction hysteresis in such knitted fabrics is still higher than in woven fabrics, it is lower compared to less elastic knitted fabrics.

The frictional interaction is still more significant in knitted fabrics due to the larger surface area and contact points created by the looped structure. However, the improved recovery characteristics mean that these fabrics can return to their original configuration more quickly after deformation, thereby potentially reducing the frictional forces acting over repeated cycles and slightly lowering the hysteresis.

Therefore, while woven fabrics, with their interlaced yarns, continue to exhibit lower friction hysteresis due to their rigidity and stability, highly elastic and well-recovering knitted fabrics show an improved performance in terms of energy dissipation and deformation recovery. This modification in the knitted structure leads to a reduction in friction hysteresis compared to less elastic knitted fabrics, although it remains higher than that of woven fabrics. Understanding these nuances is crucial for applications where the specific hysteresis behaviour of a fabric is a key consideration, especially in contexts requiring materials that can withstand repeated deformation with minimal energy loss.

## 6. Conclusions and Recommendation

Fabric, an intricate structure formed through textile processing, combines both randomness and regularity. This review explores literature on the low-stress friction and how it affects fabric mechanics at various scales. Five variables (*ε_x_*, *ε_y_*, *γ*, *K_x_*, *K_y_*) are identified and proposed as the fundamental independent variables for the pure elastic component and Coulomb friction function in a unified model of fabric mechanical properties. These variables universally apply to the tensile, shear, and bending properties of fabrics. A novel model can be found that would consider the coexistence of pure elastic and frictional hysteresis components in fabric mechanical properties, and hence establishing a multivariate nonlinear relationship for anisotropic tension, shear, and bending. In this model, the Dahl and Bliman and Sorine friction can be extended to accommodate fabric friction hysteresis. Addressing the asymmetry of mechanical properties in certain fabrics, the left and right friction stiffnesses may not be equal. The friction hysteresis model is segmented to describe the fabric’s mechanical asymmetry. Weierstrass’s function approximation theorem can then be applied, employing polynomials to approximate the purely elastic component and Coulomb friction.

By recommending a reference model that delineates the tensile, shear, and bending characteristics of fabric mechanics, we can create a unified mathematical approach. While tensile, shear, and bending properties possess distinct mechanical meanings and definition domains, they share the same mathematical regularity, and hence a unified mathematical reference model in normalized space can be formulated for fabric mechanical properties. It may be possible to use the reference model in machine learning algorithms to process fabric uniaxial measurement data with the aim to decompose and characterize the pure elastic and friction hysteresis components contained in the data. Luo and Stylios, used Kolmogorov complexity to show that the uniaxial deformation model only holds a very limited amount of algorithmic information content about the fabric [16].

In the future, it is imperative to develop instrumentation technology for the multivariate measurement of fabric deformation, aiming to address the limitation of incomplete mechanical property data obtained through uniaxial measurement systems. The successful resolution of the multivariate deformation instrument measurement technology for fabrics will enable the validation and refinement of our multivariate reference model, advancing our understanding of fabric mechanics.

The reference model should contain time-dependent aspects, such as fabric mechanics’ relaxation, creep, and material hardening, which are essential components adding complexity to fabric mechanics, and additional research is paramount to delve into these aspects.

## Data Availability

Not applicable.
